# Geospatial modeling of near subsurface temperatures of the contiguous United States for assessment of materials degradation

**DOI:** 10.1038/s41598-024-85050-3

**Published:** 2025-01-07

**Authors:** Jonathan E. Gordon, Olatunde D. Akanbi, Deepa C. Bhuvanagiri, Hope E. Omodolor, Vibha Mandayam, Roger H. French, Jeffrey M. Yarus, Erika I. Barcelos

**Affiliations:** 1 Department of Materials Science, Case Western Reserve University, Cleveland, 44106 USA; 2 Department of Computer and Data Sciences, Case Western Reserve University, Cleveland, 44106 USA; 3 Department of Earth and Planetary Sciences, Case Western Reserve University, Cleveland, 44106 USA; 4 Materials Data Science for Stockpile Stewardship: Center of Excellence, Case Western Reserve University, Cleveland, OH USA

**Keywords:** Subsurface temperature, Temperature interpolation, Geothermal gradient, Kriging, LightGBM, Machine learning, Neural networks, Geospatial modeling, Underground temperature variations, Geostatistics, Spatial data analysis, Subsurface storage design, Underground thermal variations, Temperature prediction models, State-level temperature maps, Computational methods, Computational science, Environmental sciences, Planetary science, Materials science

## Abstract

Understanding subsurface temperature variations is crucial for assessing material degradation in underground structures. This study maps subsurface temperatures across the contiguous United States for depths from 50 to 3500 m, comparing linear interpolation, gradient boosting (LightGBM), neural networks, and a novel hybrid approach combining linear interpolation with LightGBM. Results reveal heterogeneous temperature patterns both horizontally and vertically. The hybrid model performed best achieving a root mean square error of 2.61 °C at shallow depths (50–350 m). Model performance generally decreased with depth, highlighting challenges in deep temperature prediction. State-level analyses emphasized the importance of considering local geological factors. This study provides valuable insights for designing efficient underground facilities and infrastructure, underscoring the need for depth-specific and region-specific modeling approaches in subsurface temperature assessment.

## Introduction

Underground facilities, encompassing structures built below the surface for various purposes, offer numerous advantages over traditional above-ground spaces. These substructures optimize space in urban areas, provide protection from environmental elements, and offer enhanced security and stability^1^. Such facilities, including commercial buildings, waste storage facilities, data centers, and military installations, are typically constructed within the shallow subsurface area, typically considered to be the first 300 meters below the Earth’s surface^2^. However, understanding subsurface conditions, particularly temperature variations, is crucial for the design, construction, and maintenance of these facilities.

Temperature is a critical factor in the degradation of materials used in underground structures. The Arrhenius model demonstrates that temperature can accelerate chemical reactions, leading to accelerated aging and degradation of materials^3^. A temperature increase of 10 °C can double the degradation rate in polymers and the failure rate of electronic components^3,4^. Therefore, understanding subsurface temperature variations is essential for predicting and mitigating material degradation in underground facilities.

Subsurface temperatures are influenced by various factors, including mean annual surface temperatures, anthropogenic activities, and mantle heat flow^5–7^. Additional parameters such as thermal conductivity, topography, and subsurface hydrology further complicate temperature distributions^8,9^.

While subsurface temperature models have been developed for countries like Germany^10^, the Netherlands^11^, and Denmark^12^, to our knowledge, no comprehensive subsurface temperature maps covering the contiguous United States have been formally published, especially for an extended depth range.

The objective of this study is to develop subsurface temperature models of the Continental United States at various depths, ranging from ${50}\,\hbox {m}$ to ${3500}\,\hbox {m}$, by exploring and comparing different modeling techniques. We aim to demonstrate that subsurface temperatures are not uniform and that these variations must be considered in the design and planning of underground facilities. Our study employs different statistical and machine learning methods including linear interpolation, gradient boosting (LightGBM), kriging, neural networks, and a novel hybrid approach combining linear interpolation with LightGBM.

By creating temperature maps and comparing modeling techniques, we provide valuable insights for assessing the impact of temperature on the deterioration of underground infrastructures. This research contributes to the fields of geothermal modeling, materials science, and civil engineering, offering a foundation for more informed decision-making in the development and maintenance of underground facilities across the United States.

The paper is structured as follows: “Section Results” presents the results, including model comparisons and state-specific analyses. “Section Discussion” discusses the findings and their implications. “Section Methods” describes the data sources and integration and storage methodologies. Section “Methodology” outlines the methodology, including the various modeling approaches and mapping techniques, and “Section Future research directions” concludes the study with recommendations for future research.

## Results

### Model performance overview for vertical temperatures predictions

This study evaluated three main modeling approaches for estimating subsurface temperatures across the contiguous United States: Linear Interpolation, Gradient Boosting (LightGBM), and Neural Networks. Additionally, a hybrid approach combining linear interpolation with LightGBM was explored as a variation of the LightGBM method. The models were assessed using the root mean square error (RMSE), mean absolute error (MAE) and the percentage of points that fall outside specific error thresholds (1, 2, 5, and 10 °C). Table 1 provides an overview of model performance across different depth intervals.

**Table 1:** Model performance across different depth intervals.

Model type	Depth interval	Validation set size	RMSE	MAE	Outside 1 degree (%)	Outside 2 degrees (%)	Outside 5 degrees (%)	Outside 10 degrees (%)
Neural Net	0–4000 m	595,315	29.99	19.08	95.12	89.33	75.08	54.22
Neural Net	50–350 m	258,227	13.18	8.29	89.95	79.97	55.42	27.83
Linear Interpolation	0–4000 m	334,028	6.77	3.11	65.26	48.57	20.29	2.70
Linear Interpolation	50–350 m	210,618	3.18	2.12	56.25	38.11	11.53	0.70
LightGBM	0–4000 m	605,139	5.88	2.42	38.76	28.12	13.45	5.77
LightGBM	50–350 m	260,645	2.44	0.56	10.67	6.26	1.77	0.76
LightGBM (Hybrid)	0–4000 m	486,124	8.68	3.40	48.58	36.48	18.94	8.03
LightGBM (Hybrid)	50–350 m	221,785	2.61	0.68	12.05	7.46	2.12	0.87

### Linear interpolation

**Fig. 1:**
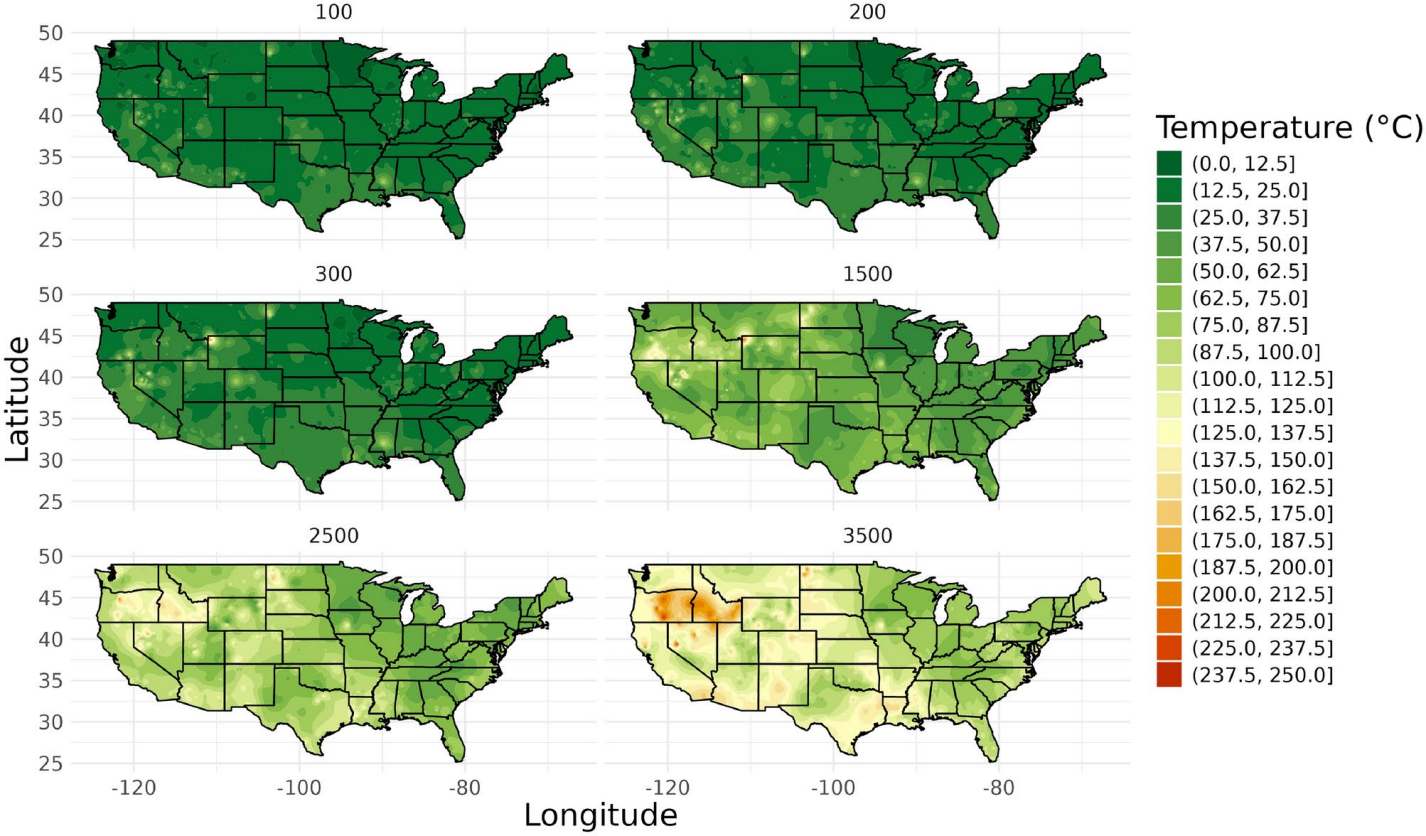
Linear Interpolation maps for all depths.

At shallow depths, linear interpolation showed moderate performance, indicating challenges in capturing local variations at shallow depths. For medium to deep depths, linear interpolation maintained relatively consistent performance. Figure 1 shows temperature maps for all depths using linear interpolation.

Table 2 provides a detailed breakdown of linear interpolation performance across different depths.

**Table 2:** Linear interpolation map performance across different depth intervals.

Depth (m)	Validation set size	RMSE	MAE	Points outside 1 degree (%)	Points outside 2 degrees (%)	Points outside 5 degrees (%)	Points outside 10 degrees (%)
100	381	11.18	4.62	56.69	43.04	19.69	12.07
200	153	13.92	6.86	71.90	60.78	36.60	21.57
300	91	14.32	7.60	75.82	57.14	41.76	13.19
1500	199	8.05	5.68	86.93	71.86	38.69	16.58
2500	175	17.76	13.62	92.00	88.57	73.14	53.71
3500	48	25.22	19.59	95.83	91.67	79.17	68.75

### Gradient boosting (LightGBM)

#### Standard LightGBM approach

**Fig. 2:**
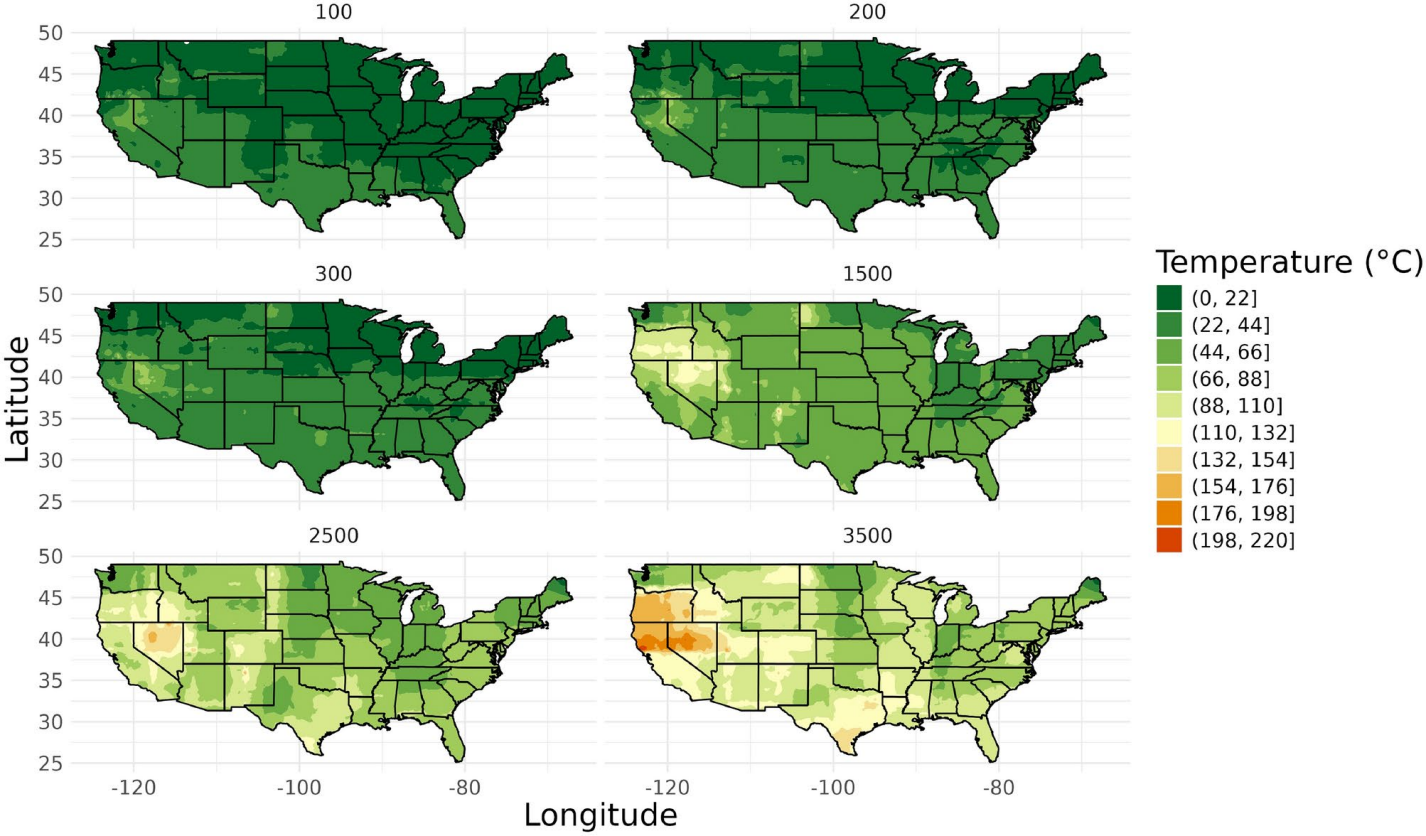
Map from LightGBM model trained on data from 0 to 4000 m.

LightGBM demonstrated superior performance at shallow depths, achieving the lowest RMSE and MAE among all models. Figure 2 illustrates the temperature maps generated using a LightGBM model trained on data from 0 to 4000 m.

Table 3 provides detailed validation results for the LightGBM each map and training approach.

**Table 3:** LightGBM model map validation results from 100 to 3500 m.

Training approach	Depth (m)	Validation set size	RMSE	MAE	Points outside 1 degree (%)	Points outside 2 degrees (%)	Points outside 5 degrees (%)	Points outside 10 degrees (%)
100–300 m	100	701	11.64	4.89	62.05	46.08	23.25	11.27
	200	282	12.64	5.53	66.31	48.58	26.24	14.54
	300	277	10.48	5.66	77.62	62.45	29.96	15.88
0–4000 m	100	663	11.99	5.65	73.15	56.86	30.47	14.48
	200	279	12.03	5.37	70.61	56.27	25.81	14.34
	300	255	10.60	6.36	82.75	68.24	33.33	19.61
	1500	847	6.10	4.22	83.83	69.30	29.52	5.90
	2500	481	12.46	9.15	91.48	83.37	61.33	35.34
	3500	198	13.54	10.04	92.42	83.33	65.66	40.40

#### Hybrid LightGBM approach

A hybrid approach combining linear interpolation with LightGBM was explored as a variation of the standard LightGBM method. This approach aimed to leverage the strengths of both gradient-based methods and machine learning techniques.

**Fig. 3:**
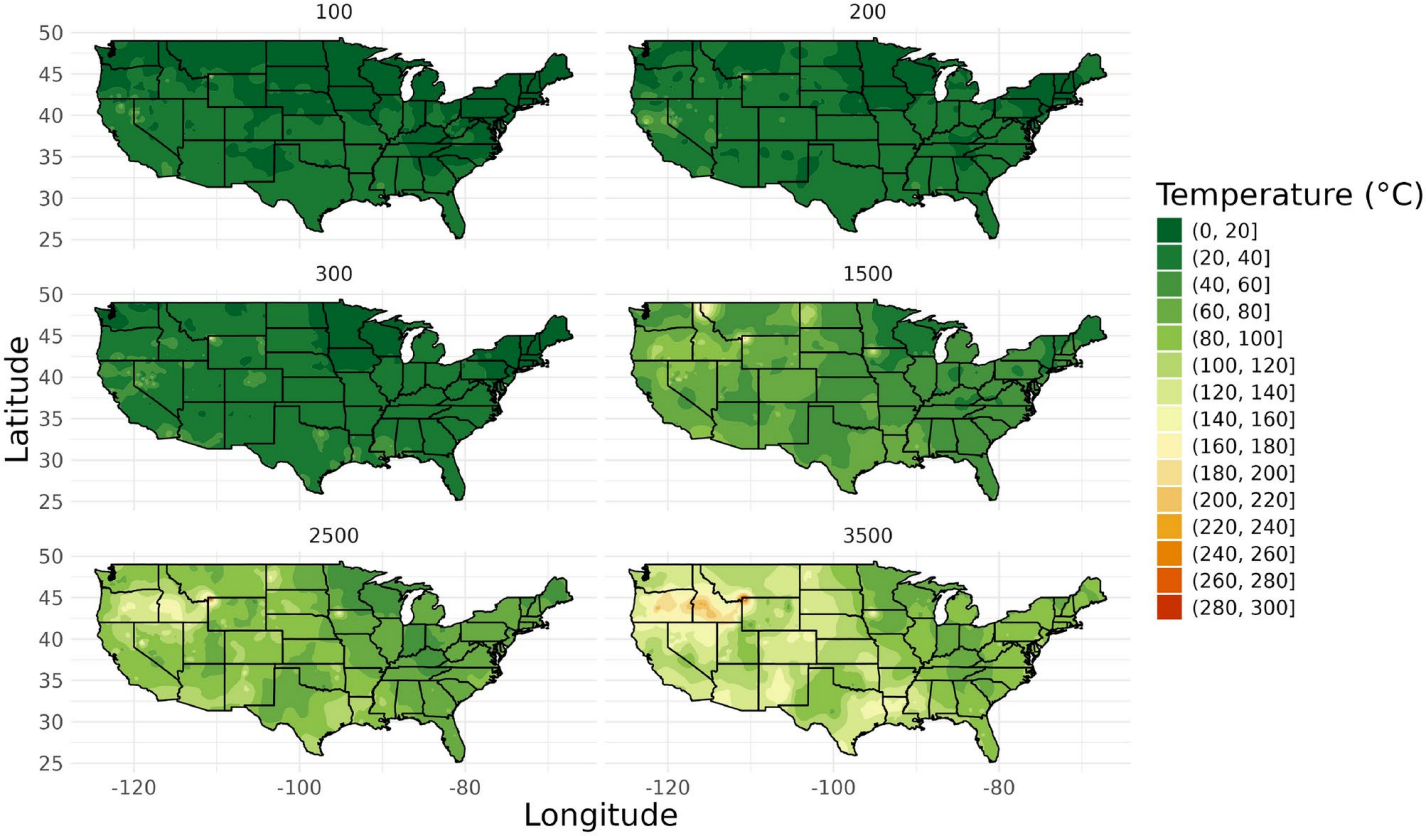
Maps from Hybrid LightGBM model trained on data from 0 to 4000 m.

The hybrid LightGBM approach showed strong performance at shallow depths outperforming the standard LightGBM, and it demonstrated better accuracy than linear interpolation. Figure 3 shows the temperature map generated using the hybrid approach model trained on data from 0 to 4000 m.

Table 4 provides detailed validation results for the hybrid model for each map and training approach.

**Table 4:** Hybrid LightGBM model map validation results.

Training approach	Depth (m)	Validation set size	RMSE	MAE	Points outside 1 degree (%)	Points outside 2 degrees (%)	Points outside 5 degrees (%)	Points outside 10 degrees (%)
100–300 m	100	406	10.43	4.28	58.87	49.01	21.18	9.36
	200	138	14.67	5.42	61.59	45.65	23.91	9.42
	300	100	10.88	5.76	76.00	62.00	28.00	13.00
0–4000 m	100	671	8.76	5.46	75.87	61.31	37.03	13.51
	200	275	9.43	6.76	88.30	73.21	46.79	28.68
	300	270	10.52	7.07	88.12	76.25	43.30	24.52
	1500	871	7.10	4.99	89.55	72.79	35.71	10.22
	2500	518	14.99	11.50	93.39	88.13	71.21	46.69
	3500	215	19.78	14.82	92.96	89.67	77.93	51.17

The hybrid approach showed better performance than linear interpolation and the standard LightGBM model. It also provided additional geological context by incorporating linear interpolation methods.

### Neural network

The Neural Network approach was explored as a potential modeling technique for subsurface temperature prediction. However, as seen in Table 1, this method showed poor performance compared to the other approaches, with high RMSE and MAE values across all depth ranges.

### Comparison of modeling approaches

To provide a comprehensive overview of the performance of different modeling approaches, we compiled a comparison table that summarizes the key metrics for each model across different depth ranges. Table 5 presents this comparison.

**Table 5:** Comparison of model approaches with average MAE values.

Approach	Depth range (m)	Overall map quality	Average map MAE (100–300 m)	Average map MAE (1500–3500 m)	Strengths	Weaknesses
Linear Interpolation	0–4000	High	6.36	9.66	Simple, fast computation	Struggles with non-linear relationships
Gradient Boosting (LightGBM)	0–4000	Moderate	5.36	6.80	Captures non-linear patterns across all depths	Requires large datasets, less interpretability
Hybrid (linear interpolation + LightGBM)	0–4000	High	5.15	8.43	Balances geological context with data-driven modeling	Computationally intensive

This comparison highlights the relative strengths and weaknesses of each modeling approach:

1. Linear Interpolation: While it offers high overall map quality and is computationally efficient, it struggles with capturing non-linear relationships in the data, particularly at shallower depths.
2. Gradient Boosting (LightGBM): This approach initially shows strong performance in capturing non-linear patterns across all depths, but it lacks geological context. It also requires larger datasets.
3. Hybrid Approach: Combining linear interpolation with LightGBM yields low average MAE values and it balances geological context with data-driven modeling. However, it is more computationally intensive.

The Neural Network approach is not included in this comparison table due to its poor performance across all depth ranges, as discussed earlier in the Results section.

These results underscore the trade-offs between model complexity, computational requirements, and predictive accuracy. The choice of modeling approach should consider the specific depth range of interest, the availability of data, and the importance of model interpretability for the given application.

### State-specific analyses

To better understand the spatial variability of subsurface temperatures across different geological settings, we conducted detailed analyses for three states: Ohio, Nevada, and Idaho. For each state, we generated two sets of temperature maps: one showing temperatures at six specific depths (100 m, 200 m, 300 m, 1500 m, 2500 m, and 3500 m), and another focusing on shallow depths (100 m, 200 m, and 300 m). All State level maps were generated using the Hybrid model.

#### Ohio

**Fig. 4:**
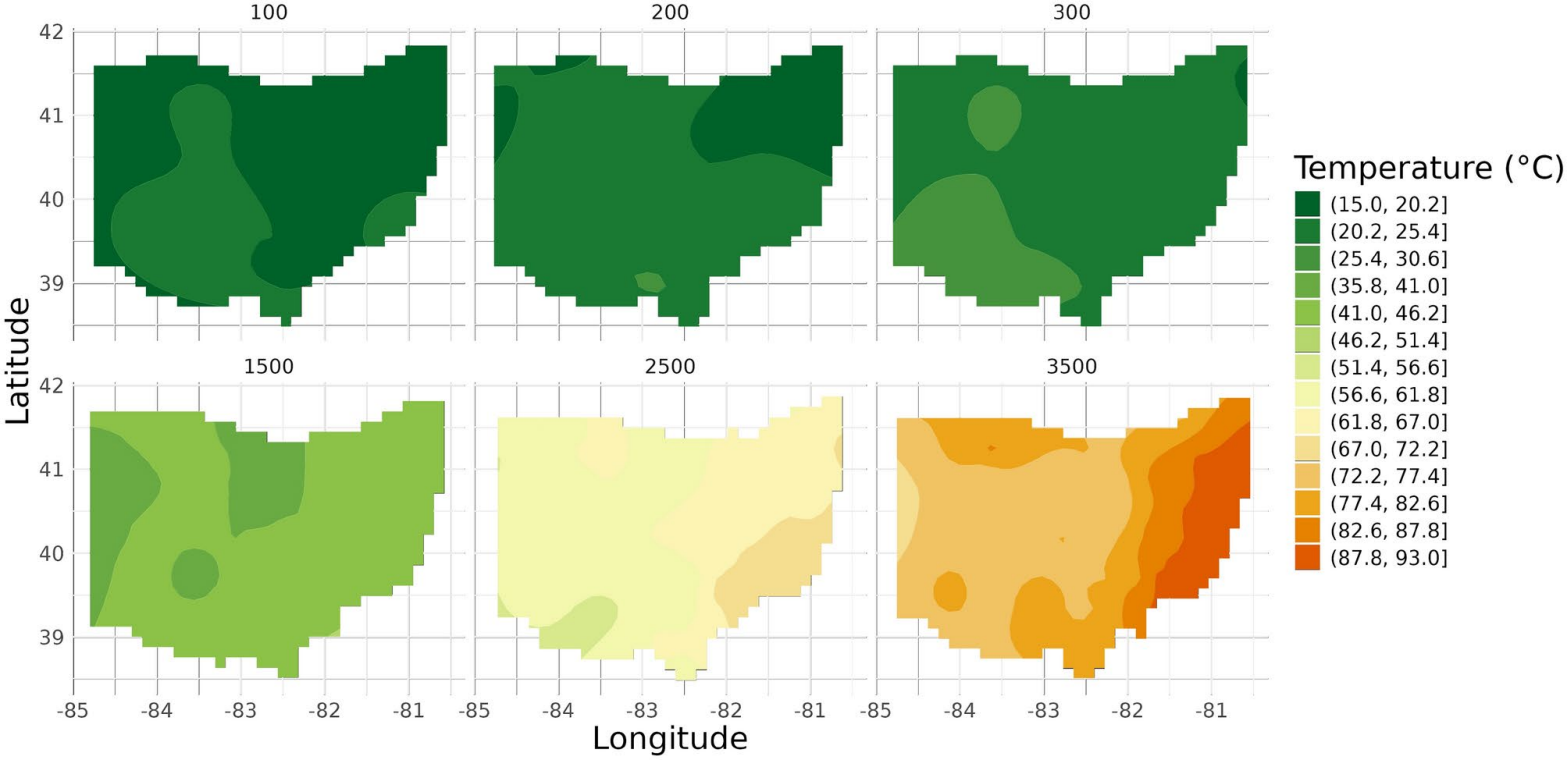
Temperature maps for Ohio at six depths (100 m, 200 m, 300 m, 1500 m, 2500 m, 3500 m).

In Fig. 4 the temperature maps for Ohio reveal relatively uniform temperature distributions, with gradual increases in temperature with depth. At shallow depths (100 m, 200 m, 300 m), Ohio still exhibits temperature variations over 10 °C which might have a large effect on the degradation rate in polymers and the failure rate of electronic components^3,4^. The maps for deeper depths (1500 m, 2500 m, 3500 m) show even more pronounced temperature gradients and potential local anomalies.

#### Nevada

**Fig. 5:**
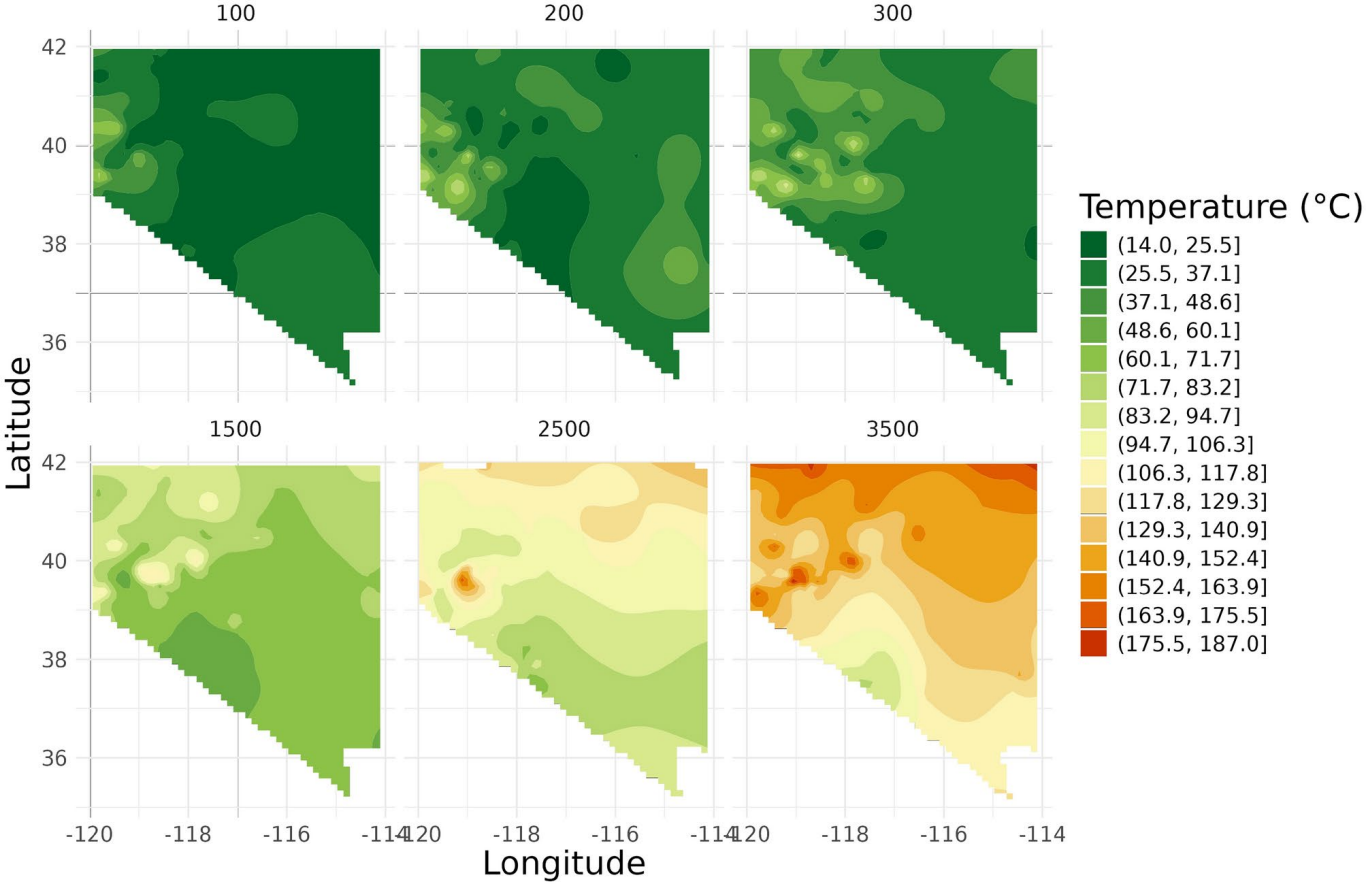
Temperature maps for Nevada at six depths (100 m, 200 m, 300 m, 1500 m, 2500 m, 3500 m).

In Fig. 5 Nevada’s temperature maps show even more heterogeneous patterns compared to Ohio, with distinct areas of higher temperatures, particularly in the northern and western parts of the state. These patterns are evident even at shallow depths (100 m, 200 m, 300 m) and become more pronounced at deeper levels (1500 m, 2500 m, 3500 m). The maps capture complex geothermal features, especially in the Basin and Range Province, across all depth ranges.

#### Idaho

**Fig. 6:**
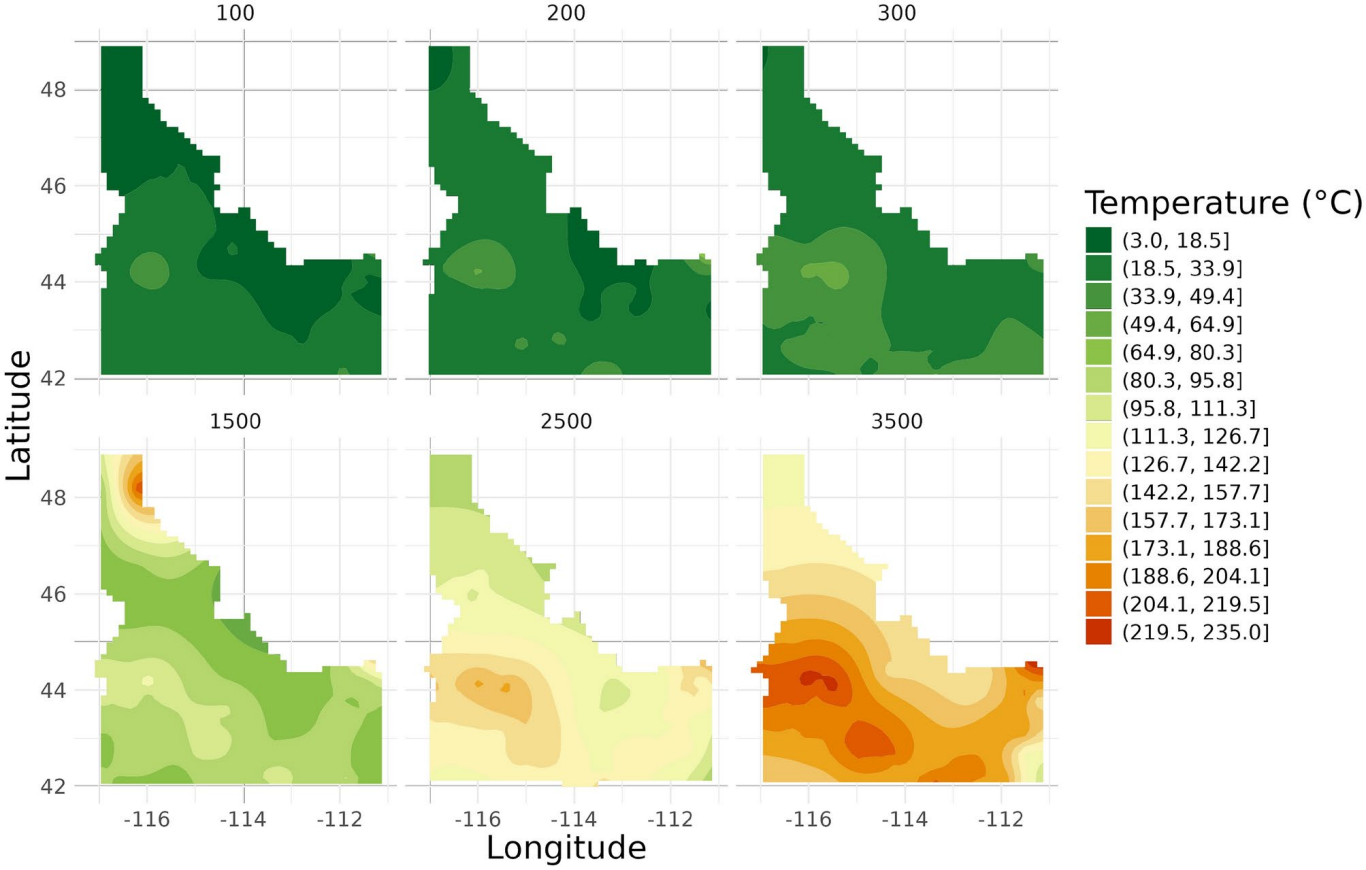
Temperature maps for Idaho at six depths (100 m, 200 m, 300 m, 1500 m, 2500 m, 3500 m).

In Fig. 6 Idaho’s temperature maps again reveal a heterogeneous patterns. At shallow depths (100 m, 200 m, 300 m), the temperature distributions show some local variations with temperature variations over 50 °C. For deeper depths (1500 m, 2500 m, 3500 m), notable temperature anomalies become apparent, particularly in the southern part of the state. These variations may correspond to known geothermal features in the region.

These state-specific analyses demonstrate the variability in temperature distributions across different geological settings and depths. They highlight the importance of considering local factors in subsurface temperature modeling. The maps reveal how temperature patterns can vary significantly not only between states but also across different depths within the same state, underlining the complex nature of subsurface thermal regimes.

## Discussion

### Interpretation of model performance

This study compared four main modeling approaches for estimating subsurface temperatures across the contiguous United States: Linear Interpolation, Gradient Boosting (LightGBM), Neural Networks, and a Hybrid Model. The results, as presented in Table 1, reveal significant differences in performance across these methods.

LightGBM demonstrated superior performance across all depth ranges, particularly excelling at shallow depths (50-350m) with the lowest RMSE (2.44 °C) and MAE (0.56 °C). This superior performance can be attributed to LightGBM’s ability to capture complex, non-linear relationships. However, the sparse data is not effectively captured by the LightGBM model, resulting in a lack of localized features and missing key information from the sparse datasets. In this case map performance can not rely on validation results alone. Geological context must be used. The map produced by LightGBM in Fig. 2 is not coherent in the geological context. Thus, while LightGBM demonstrated strong performance based on validation statistics such as MAE and RMSE, it is crucial to recognize that these metrics can be misleading when evaluating the overall model quality, particularly in complex geospatial applications like subsurface temperature modeling. Validation statistics primarily reflect how well a model fits the available data but do not necessarily capture the model’s ability to represent geophysical reality. In the case of LightGBM, the model excels at capturing non-linear relationships within the data, leading to lower error rates. However, it struggles to reflect localized geological features accurately, especially in areas where data is sparse or highly variable. As a result, the validation results alone give a false sense of accuracy.

The maps generated by LightGBM (as seen in Fig. 2) may perform well by statistical measures but fail to align with known geological expectations. This disconnect highlights the importance of not relying solely on validation statistics; instead, these metrics should be used in conjunction with geological context and domain expertise. Geophysical insights are essential for interpreting model outputs, particularly in areas where the model might oversmooth or overlook important localized anomalies that are critical for accurate subsurface predictions. Therefore, a careful assessment of the resulting temperature maps against known geological patterns is necessary to ensure meaningful and accurate predictions.

In contrast, the Linear Interpolation method showed moderate performance, with consistent results across depth ranges but less accuracy on non-linear distributions often found at shallower depths compared to LightGBM (Table 2). This result is not surprising given the complex, non-linear nature of subsurface temperature distributions, which are influenced by various geological and hydrological factors that are not easily captured by linear models. However, linear interpolation contains most of the information contained in the data sets and does well at localizing features.

The hybrid model combining linear interpolation with LightGBM, both perform well statistically at all depths (as seen in Table 1) and perform the best for the maps produced (as seen in Table 5) with the lowest averages MAE for the models trained on 100–300 m data of 5.15 °C. The hybrid model also showed promise in balancing geological context with data-driven modeling. While not outperforming the standard LightGBM model, this approach opens avenues for integrating domain knowledge with machine learning techniques.

The poor performance of the Neural Network approach across all depth ranges was unexpected and warrants further investigation. This result contradicts some previous studies that have successfully applied neural networks to geothermal modeling. The high RMSE and MAE values for the Neural Network model (Table 1) suggest potential issues with model architecture, hyperparameter tuning, or the nature of the input data that may not be well-suited for neural network processing in this context.

### Depth-dependent variations in model accuracy

A consistent trend observed across all models was the decrease in performance with increasing depth, as evident in Tables 2, 3, and 4. This trend likely reflects the increasing complexity and uncertainty of subsurface thermal regimes at greater depths.

Several factors may contribute to this depth-dependent decrease in accuracy:

1. Data scarcity: Fewer temperature measurements are typically available for deeper depths, potentially leading to increased uncertainty in model predictions.
2. Increased geological complexity: Deeper subsurface environments often exhibit more complex geological structures and thermal properties, which may not be fully captured by our models.
3. Influence of deep geological processes: At greater depths, factors such as radiogenic heat production and mantle heat flow become more significant, introducing additional variables that our models may not adequately account for.
4. Extrapolation errors: Models trained primarily on shallower data may struggle to accurately extrapolate to deeper depths where the relationships between variables may change.

The hybrid approach, combining linear interpolation with LightGBM, showed promise in mitigating some of these depth-related issues (Table 4), suggesting that incorporating geological knowledge into machine learning models could be a fruitful direction for future research.

### Comparison with existing literature

Our findings both corroborate and extend previous studies on subsurface temperature modeling in the United States. The observed spatial variations in temperature patterns align with the broad trends reported in earlier works, such as the comprehensive heat flow study^13^. However, our study provides higher resolution and depth-specific temperature maps, offering more detailed insights into local variations.

The superior performance of the Hybrid model, particularly at shallow depths, represents a notable advancement in the field. Although previous studies have mainly relied on interpolation methods or simpler statistical approaches, our results demonstrate the potential of machine learning techniques to improve the accuracy of subsurface temperature predictions.

Our observation of decreasing model performance with depth is consistent with challenges reported in other deep geothermal studies^14^. However, our hybrid approach, combining linear interpolation with LightGBM, offers a novel solution to this common problem, potentially bridging the gap between data-driven and physics-based modeling approaches.

### Implications for geothermal resource assessment and infrastructure planning

The high-resolution temperature maps and model performance metrics presented in this study have significant implications for both geothermal resource assessment and underground infrastructure planning.

For geothermal energy development, our results provide a more nuanced understanding of temperature distributions at various depths. The superior performance of the LightGBM model at shallow depths (Table 3) is particularly relevant for the planning of ground source heat pump systems and shallow geothermal applications.

In the context of underground infrastructure planning, our findings underscore the importance of considering depth-specific temperature variations. The observed decrease in model accuracy with depth (Table 5) highlights the need for cautious interpretation of temperature predictions for deep underground structures. This information is crucial for the selection of appropriate materials and the design of thermal management systems in deep underground facilities.

Moreover, the spatial variability revealed in our state-specific analyses emphasizes the need for localized approaches in infrastructure planning. The contrasting temperature patterns observed in Ohio, Nevada, and Idaho demonstrate that a one-size-fits-all approach to underground construction and material selection may not be appropriate across different geological settings.

### Future research directions

Based on our findings and the limitations identified, we propose several directions for future research:

1. Integration of additional data sources: Incorporating data on groundwater flow, detailed geological structures, and surface heat flux could enhance the accuracy of temperature predictions, especially at greater depths.
2. Temporal modeling: Extending the current approaches to include time-series analysis could provide insights into how subsurface temperatures change over time, which is crucial for long-term infrastructure planning and climate change studies.
3. High-resolution regional studies: Conducting more detailed analyses in specific regions of interest, using higher resolution data where available, could provide valuable insights for local geothermal resource assessment and infrastructure planning.
4. Uncertainty quantification: Developing methods to quantify and visualize the uncertainty in temperature predictions would enhance the utility of these models for decision-making processes.
5. Cross-validation with new drilling data: As new subsurface temperature data becomes available from ongoing drilling projects, cross-validating and refining our models will be crucial for improving their accuracy and reliability.

In conclusion, while our study represents a significant step forward in subsurface temperature modeling, it also highlights the complexity of this field and the numerous opportunities for further research and improvement. The integration of advanced machine learning techniques with geological knowledge promises to enhance our understanding and prediction of subsurface thermal regimes, with far-reaching implications for geothermal energy development, underground infrastructure design, and our broader understanding of Earth’s thermal structure.

## Methods

This section provides an overview of the data sources employed, along with the methodologies used for data ingestion, storage, and standardization, using the Common Research Analytics and Data Lifecycle Environment (CRADLE). Developed on a Distributed and High-Performance Computing (D/HPC) platform, CRADLE’s framework has been discussed in previous works^15–18^. Two main sources of data were employed in this work : Well data from public sources and data extracted from Nathenson et al.^19^ work.

### Well data from publicly available sources

**Fig. 7:**
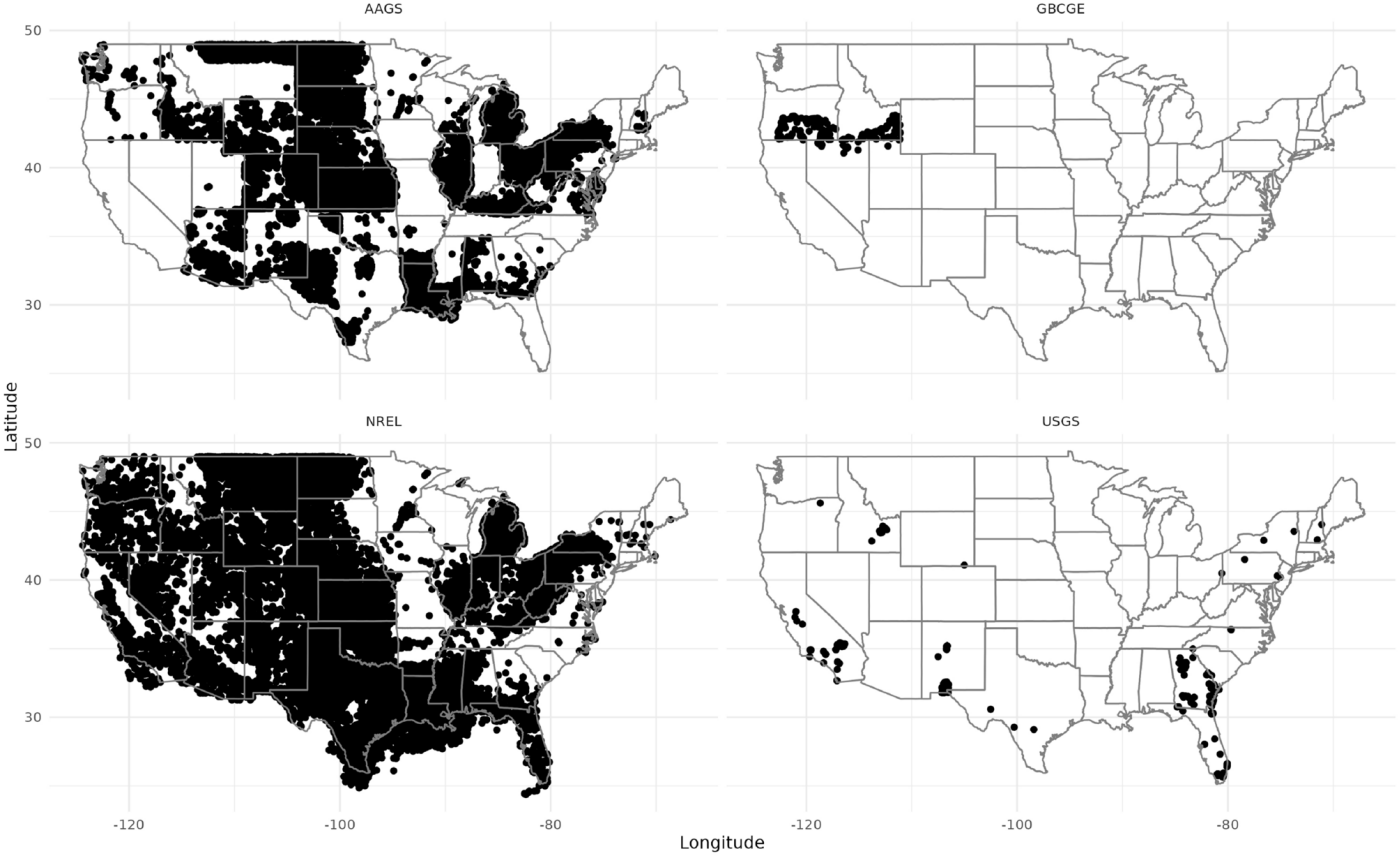
Locations of wells from each data set. Each dot represents a location of a well.

Temperature and depth data were downloaded from four public sources: water well records retrieved from the United States Geological Survey (USGS)^20^, oil and gas well data obtained from the National Renewable Energy Laboratory (NREL) Geothermal Prospector^21^, borehole temperature data from the American Association for Geodetic Surveying (AAGS)^22^, and digitized temperature well logs from the Great Basin Center for Geological Energy’s (GBCGE) Subsurface Database Explorer and API^23^. Figure 7 summarizes the main data sources with their respective number of entries and wells.

**Fig. 8:**
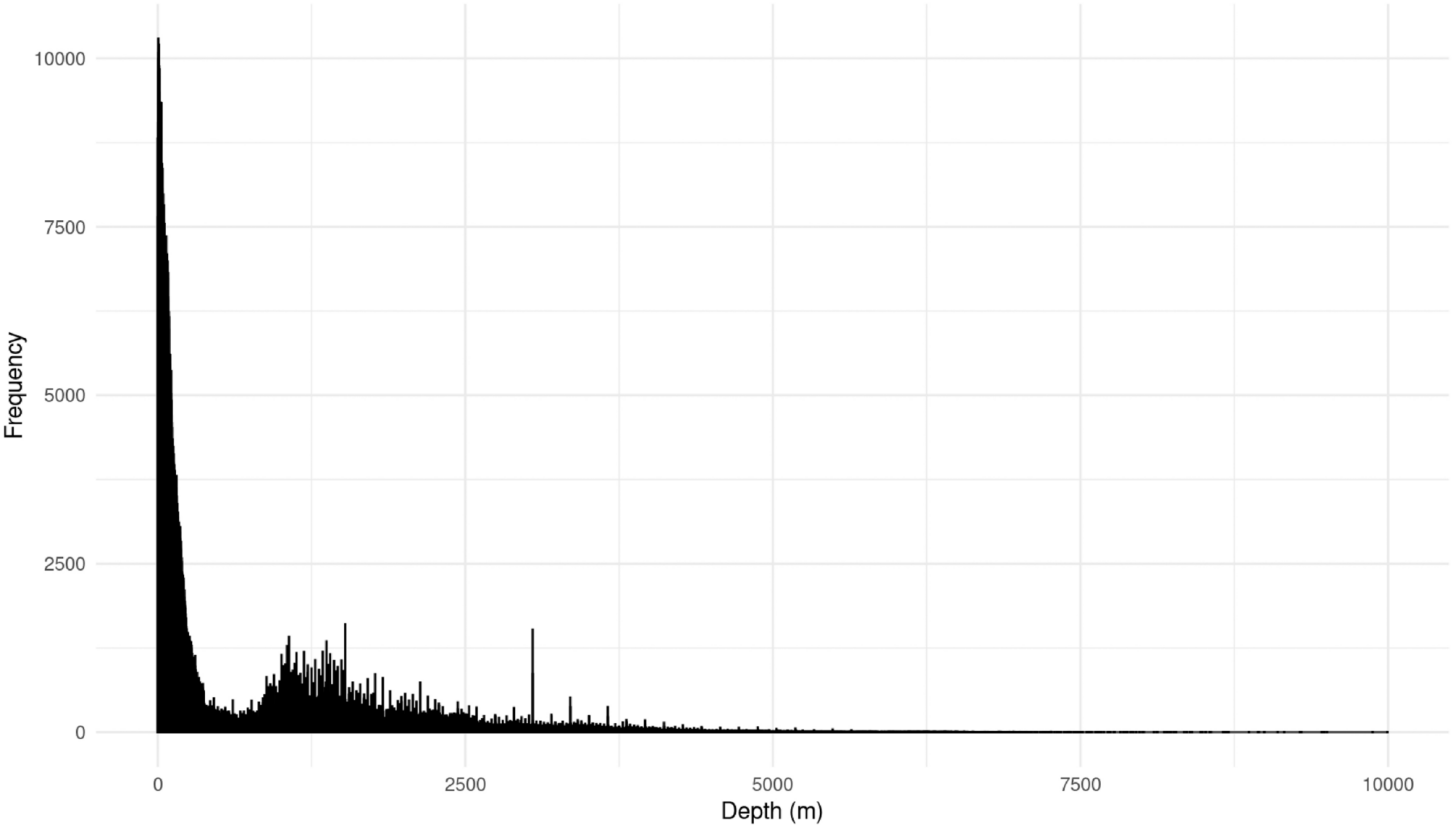
Histogram illustrating the data distribution for the all the 445,666 wells combined from the four data sources.

The datasets from NREL, USGS, and AAGS were downloaded between September of 2021 and March of 2023. The GBCGE data set was collected using the GBCGE Subsurface Database API in July of 2024. The combined data sets include 445,666 Wells and 1,978,655 data points across all 48 contiguous states. Table 6 shows the size of each data set and Fig. 8 shows the distribution of the data at by depth.

**Table 6:** Data summary of wells in different datasets.

Dataset	Total data entries	Unique wells
AAGS	299,772	271,980
GBCGE	99,349	501
NREL	113,206	97,421
USGS	1,587,781	1,203

Figure 8 shows that a significant portion of the data points are shallow, with approximately 69.8% of the depths being less than 1000 meters and about 37.5% of the data points falling under 100 meters. A smaller proportion of the dataset extends to greater depths, with 95.1% of the observations being less than 3000 meters deep.

### Gradient data extracted from from Nathenson et al.^19^

Initially, 284 gradient values were extracted from Nathenson et al. (1987)^24^. These values were kriged using the RGeostats package to create a continuous 2D geothermal gradient map for the contiguous United States. These gradients were used in Eq. 1 for linear interpolation of temperatures.

### Data integration, ingestion, and storage

All datasets underwent a cleaning and integration process before being employed to generate estimated temperature maps for the contiguous United States. Data collected from USGS and GBCGE were structured in a well-log format, where each well had multiple depths and corresponding temperature measurements. This format proved ideal for creating temperature maps at different depths; however, the temperature data from USGS and GBCGE were not well dispersed throughout the US.

In contrast, data obtained from NREL and AAGS had better geographical coverage (Fig. 7) but contained only bottom hole temperature (BHT) measurements, resulting in a single depth measurement paired with its corresponding temperature measurement.

Due to these factors, specific cleaning and integration methodologies were implemented to merge these datasets. This was done to leverage the geographical coverage provided by the NREL dataset with the experimental measurements obtained at multiple depths per well from the USGS dataset.

For NREL data with only bottom hole temperature (BHT) measurements, a linear interpolation method was employed to estimate temperatures at the desired depths, effectively converting the data into a well-log format^25^.

The observed and extrapolated temperature data at various depths from all datasets were restructured to adhere to a standardized format, including metric units and relevant features shared between the datasets, such as temperature and depth measurements, location coordinates, state labels, and more. The integrated dataset was then prepared for use in the various modeling approaches, including linear interpolation, LightGBM, neural networks, and the hybrid approach combining linear interpolation with LightGBM.

## Methodology

### Modeling approaches

This study employs several modeling approaches to estimate subsurface temperatures across the contiguous United States, including Ordinary Kriging, Linear Interpolation, LightGBM, DNN, and a hybrid approach. Table 7 summarizes the models and their respective direction of predictions. The vertical direction means that predictions are made at multiple depths while the horizontal direction is related to horizontal spatial prediction.

**Table 7:** Models used with corresponding estimation direction.

Model	Direction
Linear Interpolation	Vertical
Neural Networks	Vertical
LightGBM	Vertical
Hybrid Approach	Vertical
Kriging	Horizontal

#### Ordinary Kriging

Ordinary Kriging is a geostatistical technique used for spatial interpolation that takes into account both the distance and degree of variation between known data points. The RGeostats package was used to perform variogram analysis and spatial dependence of the model for temperature estimation. Ordinary Kriging was applied to create smooth predictive surfaces, accounting for local spatial correlations.

#### Linear interpolation

Maps are created traditionally using linear interpolation with temperature gradients.

Linear interpolation estimates unknown values between two known data points. In this context temperature gradient data were employed^24^ to provide a slope and a known temperature-depth value to create an equation to model temperature at every well:

1 $$\begin{aligned} T = T_0 + G(z - z_0) \end{aligned}$$

where $T_0$ and $z_0$ are the known depth and temperature values, *G* is the gradient at that well, and *z* is the depth value where temperature is to be interpolated.

#### LightGBM

LightGBM is a gradient boosting framework that uses tree-based learning algorithms. It is highly efficient for handling large-scale data and high-dimensional features. LightGBM has recently been used to model subsurface temperatures in several studies^26,27^. The LightGBM model was implemented using the LightGBM package^1^.

**Fig. 9:**
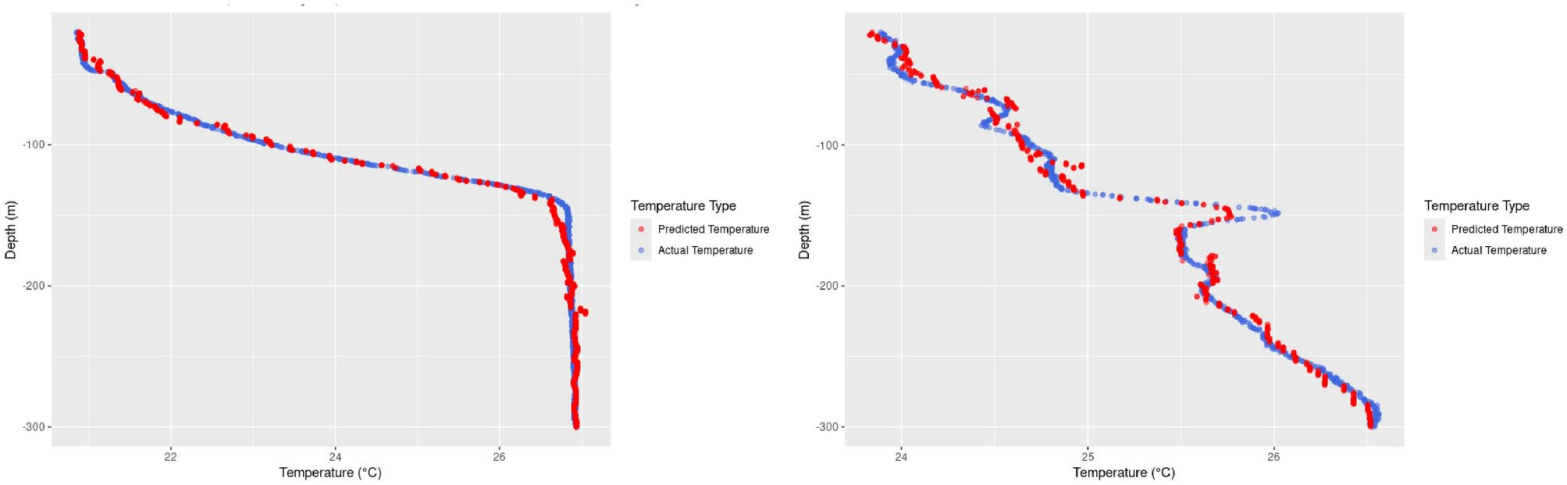
Actual versus predicted temperature of Well using LightGBM model for two wells in Florida. Right in Duval County Florida ID-0662 J0801 Well, and left side in Lake Nona OUC well in Orange County, Florida.

LightGBM excels at capturing non-linear relationships and interactions between features as seen in Fig. 9, allowing it to model more complex temperature behavior at shallower depths. This leads to improved accuracy over simpler methods like linear interpolation. LightGBM also generates a model that allows for temperature to be interpolated at locations where there is no well data, unlike linear interpolation.

Figure 9, showing two wells with non linear behavior, illustrates how LightGBM learns the more chaotic fluctuations in temperature, particularly in regions where linear models would oversimplify the behavior.

#### Deep neural networks

Deep Neural Network (DNN) models are inspired by the brain’s neural networks and are capable of modeling complex non-linear relationships. The neural network was implemented using the keras package^28^, with a focus on capturing deeper interactions between spatial coordinates and depth.

#### Hybrid approach

The hybrid approach combines the strengths of both Linear Interpolation and LightGBM by first using Linear Interpolation to generate projected temperatures at specific depths across all wells. These projected values serve as input data for training the LightGBM model. By doing this, the model benefits from both the geologically informed projections of gradient data and the flexible, non-linear relationships captured by LightGBM.

### Gradient mapping

The 284 gradient values extracted from Nathenson et al.^24^ were kriged to generate a gradient map, which was subsequently used to determine the gradients at each well. These gradients provided the necessary slopes for the linear interpolation process. The gradient map was generated using the vario.calc() function from the RGeostats package, which employed a lag distance of one degree and included a total of eight lags. A variogram model was fitted using the spherical and nugget functions from the model.auto() function within the same package. The grid cell size for the final gradient map was set to 0.125 degrees.

After generating the continuous gradient map, the nearest grid cell on the 2D gradient map to each well was identified using a nearest-neighbors search. The gradient value from the nearest cell was then used as the variable in Eq. 1 to calculate the temperature at the desired depth.

### Model methodology

In this study models had two main functions horizontal and vertical interpolation. Kriging was utilized for horizontal interpolation, filling in the gaps between wells, while vertical interpolation within each well was carried out using linear regression, LightGBM, and DNN models. Although LightGBM and DNN are capable of predicting values beyond known wells, Kriging was still applied to interpolate between wells to ensure a more accurate representation of the spatial distribution of temperatures. Table 7 summarizes the models with the corresponding depths and directions.

#### Model training

For each approach, the data was split into train, test, and validation sets using an 56/14/30 ratio, and a training matrix was constructed using latitude, longitude, and depth as input features. The models were optimized with cross-validation against the test set. Computation-intensive tasks, such as DNN training and LightGBM model fitting, model prediction and others were parallelized using the future.apply package^29^, reducing runtime across large spatial datasets.

#### Model selection

We evaluated each approach for performance across various depth intervals, primarily focusing on ranges from 50 to 350 meters and 0 to 4000 meters. The evaluation metrics used included Root Mean Squared Error (RMSE), Mean Absolute Error (MAE), and the percentage of points with predictions falling outside of 1, 2, 5, and 10 degrees from the true values. This comparison allowed us to assess how well each model performed across different depth intervals and choose which approaches would be most effective for generating accurate subsurface temperature predictions. The models were then selected to be used for the generation of maps.

### Mapping methodology

All datasets were cleaned and harmonized to ensure consistent formats and units. Missing or erroneous temperature measurements were filtered out, each well was given a local unique ID and all data was aggregated. The selected models were then used to predict temperature values at specified depths for well locations with nearby data.

Following, the predicted data were kriged to create a map. Due to the computational difficulties of kriging, a random sample of up to 150 points were selected from each state when performing kriging. Variograms were created for each map with the vario.calc() function from the RGeostats package to estimate temperatures at unsampled locations based on known spatial correlations as a subsequent step. A variogram model was fitted using the spherical function from the model.auto() function also from RGeostats. The grid cell size for the final gradient map was set to 0.125 degrees.

To spatially interpolate the gradients, a moving neighborhood approach was used. This neighborhood was set up with a search radius of 5 units latitude-longitude and a limit of 1250 points within each neighborhood. All function parameters were empirically determined to best fit the dataset.

Finally, the kriged data was plotted using the ggplot package^30^.

Additionally, we performed state-specific analyses to observe temperature variation patterns on a closer scale. Three states (Nevada, Ohio, and Idaho) were selected for detailed comparison of the different modeling approaches. These maps were generated by filtering the kriged data by state before mapping.

### Validation methodology

Validation was conducted using RMSE and MAE to compare predictions with observed well data in the validation set to provide an error for each map. All points within 5 m of the map depth in the validation set were averaged for each well. Then a nearest neighbor search found the closest grid point to each validation point, and the residuals were calculated to determine the validation statistics.

## Data Availability

The datasets in this study can be found at the following hyperlinks: USGS dataset, GBCGE dataset, AAGS dataset, and NREL dataset. Cleaned and processed versions of these datasets are available upon request from the corresponding authors.

## References

[1] Shi, Y. *et al.**LightGBM: Light Gradient Boosting Machine*. R package version 4.5.0.99 (2024).

[2] Zaini, F., Hussin, K., Jamalludin, N. A. & Zakaria, S. R. A. The principle of depth for underground land development: A review. *Jurnal Teknologi*. 10.11113/jt.v75.5275 (2015).

[3] Leenson, I. A. Old rule of thumb and the Arrhenius equation. *J. Chem. Educ.* **76**, 1459. 10.1021/ed076p1459 (1999).

[4] Bayle, F. & Mettas, A. Temperature acceleration models in reliability predictions: Justification & improvements. In *2010 Proceedings—Annual Reliability and Maintainability Symposium (RAMS)*, 1–6. 10.1109/RAMS.2010.5448028 (2010).

[5] Slagstad, T., Midttømme, K., Ramstad, R. & Slagstad, D. Factors influencing shallow (< 1000 m depth) temperatures and their significance for extraction of ground-source heat, vol. 11, 99–109 (2008).

[6] Pollack, H. N. & Chapman, D. S. Mantle heat flow. *Earth Planet. Sci. Lett.* **34**, 174–184. 10.1016/0012-821X(77)90002-4 (1977).

[7] Loria, A. F. R. The silent impact of underground climate change on civil infrastructure. *Commun. Eng.* **2**, 1–12. 10.1038/s44172-023-00092-1 (2023).

[8] Chulick, G. S. & Mooney, W. D. Seismic structure of the crust and uppermost mantle of North America and adjacent oceanic basins: A synthesis. *Bull. Seismol. Soc. Am.* **92**, 2478–2492. 10.1785/0120010188 (2002).

[9] Štulc, P. Combined effect of topography and hydrogeology on subsurface temperature–Implications for aquifer permeability and heat flow. A study from the Bohemian Cretaceous basin. *Tectonophysics* **284**, 161–174. 10.1016/S0040-1951(97)00171-6 (1998).

[10] Agemar, T., Schellschmidt, R. & Schulz, R. Subsurface temperature distribution in Germany. *Geothermics* **44**, 65–77. 10.1016/j.geothermics.2012.07.002 (2012).

[11] Bonté, D., van Wees, J. D. & Verweij, J. M. *Subsurface Temperature of the Onshore Netherlands: New Temperature Dataset and Modelling* (Cambridge University Press, 2012).

[12] Balling, N. et al. Geothermal measurements and subsurface temperature modelling in Denmark. *GeoSkrifter* **16**, 172 (1981).

[13] Blackwell, D. et al. *Temperature-at-depth maps for the conterminous us and geothermal resource estimates*. Tech. Rep., Southern Methodist University Geothermal Laboratory, Dallas, TX (United States) (2011).

[14] Jiang, G.-Z. et al. Compilation of heat flow data in the continental area of china. *Chin. J. Geophys.* **59**, 2892–2910 (2016).

[15] Hu, Y. et al. A nonrelational data warehouse for the analysis of field and laboratory data from multiple heterogeneous photovoltaic test sites. *IEEE J. Photovolt.* **7**, 230–236. 10.1109/JPHOTOV.2016.2626919 (2017).

[16] Khalilnejad, A. et al. Automated pipeline framework for processing of large-scale building energy time series data. *PLoS ONE* **15**, e0240461. 10.1371/journal.pone.0240461 (2020). 33259504 10.1371/journal.pone.0240461 PMC7707597

[17] Nihar, A. *et al.**Accelerating Time to Science Using Cradle: A Framework for Materials Data Science.* (IEEE, Goa, India). 10.1109/HiPC58850.2023.00041. (2023).

[18] Akanbi, O. D. et al. Integrating multiscale geospatial analysis for monitoring crop growth, nutrient distribution, and hydrological dynamics in large-scale agricultural systems. *J. Geovisual. Spatial Anal.* **8**, 9. 10.1007/s41651-023-00164-y (2024).

[19] Nathenson, M. & Guffanti, M. Geothermal gradients in the conterminous United States. *J. Geophys. Res. Solid Earth* **93**, 6437–6450. 10.1029/JB093iB06p06437 (1988).

[20] USGS—GeoLog Locator. https://webapps.usgs.gov/GeoLogLocator/#!/search.

[21] Innovative Data Energy Applications. https://maps.nrel.gov/?da=geothermal-prospector.

[22] Geothermal Data Repository (GDR). https://gdr.openei.org/.

[23] Mlawsky, E. & Ayling, B. GBCGE subsurface database explorer and APIs. 10.15121/1987556 (2020).

[24] Nathenson, M., Manuel & Guffanti, Marianne. Compilation of Geothermal-Gradient in the Continuous United States. Open-File Report 87-592, United States Geological Survey (1987).

[25] James, G., Witten, D., Hastie, T., Tibshirani, R. & Taylor, J. *An Introduction to Statistical Learning: With Applications in Python. Springer Texts in Statistics* (Springer International Publishing, 2023).

[26] Taddia, G. & Gizzi, M. *Integration of Machine Learning Methodologies for Enhancing Subsurface Geothermal Resource Analysis and Development*. Electronic, Politecnico di Torino, Turin, Italy (2023). Corso di laurea magistrale in Petroleum and Mining Engineering (Ingegneria Del Petrolio e Mineraria), 68.

[27] Su, H. et al. Super-resolution of subsurface temperature field from remote sensing observations based on machine learning. *Int. J. Appl. Earth Obs. Geoinf.* **102**, 102440. 10.1016/j.jag.2021.102440 (2021).

[28] Chollet, F. *et al.* Keras. https://keras.io (2015).

[29] Bengtsson, H. A unifying framework for parallel and distributed processing in R using futures. *R J.* **13**, 208–227. 10.32614/RJ-2021-048 (2021).

[30] Wickham, H. *ggplot2: Elegant Graphics for Data Analysis* (Springer, 2016).

